# Chlamydial infection among patients attending STD and genitourinary clinics in Taiwan

**DOI:** 10.1186/1471-2458-7-120

**Published:** 2007-06-25

**Authors:** Kow-Tong Chen, Shou-Chien Chen, Chien-Chou Chiang, Lan-Hui Li, Li-Hui Tang

**Affiliations:** 1 Department of Public Health, College of Medicine, National Cheng- Kung University, Tainan, Taiwan; 2 Department of Bioengineering, Tatung University, Tiapei, Taiwan; 3 Department of Family Medicine, Zhongxiao Branch, Taipei City Hospital, Taipei, Taiwan; 4 Taipei City STD Control Center, Taipei, Taiwan; 5 Center for Disease Control, Department of Health, Taipei, Taiwan

## Abstract

**Background:**

The main objective of this study is to examine the epidemiology of *Chlamydia trachomatis *(CT) infection amongst patients (473 men, 180 women) seen two hospitals in Taiwan.

**Methods:**

Between July 2004   and June 2005, a total of 653 patients provided first-void urine samples for examination of CT using PCR assay.

**Results:**

The overall prevalence of CT infection was 18.4% (95% confidence interval [CI] 17.3–19.5). Prevalence for men and women were 16.7 % (95% CI 15.3–18.0%) and 22.8% (95% CI 17.5–28.1%), respectively. Age group-specific prevalence was 25.7% (95% CI 22.5–28.9%) in < 20 year olds, 23.5% (95% CI 20.3–26.7%) in 20–24 year olds, 22.3% (95% CI 18.9–25.7%) in 25–30 year olds, and 11.5% (95% CI 10.3–12.7%) in > 30 year olds. Independent risk factors for chlamydial infection included younger age (aged ≤ 30 years) (adjusted odds ratio [AOR] = 2.44; 95% CI 1.52–3.84; *p *< 0.001), inconsistent condom use (AOR = 2.01; 95% CI 1.32–3.06; *p *< 0.001), being symptomatic (dysuria, urethral discharge) at the time of testing (AOR = 1.84; 95% CI 1.21–2.80; *p *< 0.001), and having *N. gonorrhoeae *infection (AOR = 3.82; 95% CI 2.20–6.58; *p *< 0.001).

**Conclusion:**

Genital chlamydial infection is an important sexually transmitted disease in Taiwan. Young Taiwanese persons attending a STD clinic should be screened for CT infection and counselled on condom use.

## Background

Young, sexually active people are at high risk for genital infection by *Chlamydia trachomatis *(CT) [1,2]. It is estimated that 340 million new cases of common bacterial and protozoal sexually transmitted infections occur annually worldwide [3]. An increase in sexually risky behaviour amongst young people and an associated subsequent increase in the prevalence of CT have been reported [4,5]. Chlamydial infections can cause severe complications in untreated infected women such as pelvic inflammatory diseases (PID), ectopic pregnancy, tubal infertility and abdominal pain [6]. Serious sequelae among men with untreated infection include urethritis, prostatitis, epididymitis and Reiter syndrome [6]. Active CT infection is an important risk factor facilitating sexual transmission of HIV infection [7]. Most women (> 50%) infected with CT remain symptom-free, and for men, the rate of asymptomatic chlamydia infection is higher than for symptomatic gonorrhea infection [8-10]. Early diagnosis and early treatment of affected individuals are the types of strategies necessary to prevent the development of sequelae and to reduce transmission [6]. In Taiwan, although the incidence and prevalence of CT infection is rising [11], only limited information is available. The aim of this study was therefore to examine the epidemiology of genital chlamydia infection amongst patients attending STD and genitourinary clinics in Taiwan.

## Methods

### Patients

The pool of patients included all patients attending two outpatient clinics between July 2004 and June 2005; one group was investigated at the Taipei City STD Control Center clinic (northern Taiwan) and another group was studied at genitourinary outpatient clinic at a Kaohsiung primary care service (southern Taiwan).

Taipei and Kaohsiung are two large metropolitan areas in Taiwan. Only those patients who met the following criteria were included for study: aged> 17 years, sexually active (reported to have sexual intercourse in the last 60 days) provided a urine specimen and no antibiotic use in the preceding 15 days. Approval was obtained from the Ethics Committee of Taipei City STD Control Center. After signing an informed consent form, patients were given a self-administered questionnaire regarding limited demographic information, symptoms of urethritis (dysuria, urethral discharge) and condom use during the last intercourse.

A patient was classified as "symptomatic" if the patient reported having one of the symptoms (dysuria, urethral discharge) and as "non-symptomatic" if neither of these symptoms were reported.

Subsequently, a genital examination was performed by a doctor and blood samples were tested for *Treponema pallidum *and HIV antibodies. Each participant provided a first void urine specimen that was tested for CT, *Neisseria gonorrhoeae*, and *Trichomonas vaginalis*. Those individuals who had a positive result for the test were informed of this outcome by a personal telephone call, at which time a medical appointment was scheduled at the STD clinic for both the patient and partner(s).

### HIV serodiagnosis and STD diagnosis

The Amplicor PCR test for *Chlamydia trachomatis/Neisseria gonorrhoeae *(PCR Roche Diagnostic Corp, Indianapolis, IN, USA) was performed on all individual samples according to the manufacturer's instructions for processing urine specimens [12]. To diagnose *T. vaginalis*, wet-mount microscopy and culture in Whittington media (HiMedia Laboratories, Mumbai, India) were performed on urine specimens. The Amplicor positive urine samples were stored at -20°C for further analysis. The Rapid Plasma Reagin (RPR; Spinreact, Girona, Spain) or the Venereal Disease Research Laboratory (VDRL) tests were used for syphilis screening [13], with confirmation by the *T. Pallidum *haemagglutination assay (TPHA; Gesellschaft fur Biochemica and Diagnostica, Wiesbaden, Germany) following the manufacturer's instructions. Sera positivity by both the RPR/VDRL test (at 1:8 dilution) and the TPHA test were considered to be indicative of active syphilis. For HIV detection, the double-ELISA test (Biosign kits, Princeton BioMeditech, Princeton, NJ, USA) and western blot assays (Genelabs Diagnostic, Redwood City, CA, USA) were performed.

### Statistical analysis

For analysis, chlamydial infection was defined as a urine sample that was positive by the diagnostic Amplicor PCR test. Statistical analysis was conducted using SPSS software for Windows (version 10.0; SPSS). The prevalence of genital chlamydia infection was calculated with 95% confidence intervals. All variables were analyzed using the Chi-square test or Fisher's exact test for categorical variables. All variables that revealed significant results in the context of univariate analysis were subsequently entered stepwise into a multiple logistic regression analysis procedure, for which all measured effects were controlled for by means of controlling the influence of all other variables at the time of testing. Significance was defined by an alpha level of 0.05 or less.

## Results

### Characteristics of all patients

During the period from July 2004 to June 2005, a total of 687 patients were eligible for the study. Among these, 653 (95.1%) patients met the inclusion criteria and were enrolled the study. The characteristics of the study population are indicated in Table 1. The age of study patients ranged from 17 to 50 years and the median age was 28 years. Seventy-two percent were males and 28% were females. The most-common age group was ≤ 30 years old (55%); 75% were single and 25% were married; the prevalence of CT among symptomatic patients was 23%; 49% were symptomatic at the time of testing (dysuria or discharge); 45% not always using condoms during sexual intercourse; 90% were heterosexual and 10.0% were homosexual/bisexual.

Of the 653 samples, the prevalences of *C. trachomatis*, *N. gonorrhoeae, T. Pallidum*, HIV, and *T. Vaginalis *infection were 18.4% (120/653), 11.2% (73/473), 5.7% (37/653), 1.2% (8/653) and 0.9% (6/653), respectively. No pathogens were identified in urine obtained from 72.3% of the study subjects.

**Table 1:** Demographic characteristics of 653 subjects by status of *C. trachomatis *infection, 2004 – 2005, Taiwan

Variables	Chlamydia (+)	Chlamydia (-)	Total^b^
	N = 120	N = 533	N= 653
Gender			
Male	79 (16.7)	394 (83.3)	473 (72)
Female	41 (22.8)	139 (77.2)	180 (28)
Age group (years)*			
< 20	35 (25.7)	101 (74.3)	136 (21)
20–24	28 (23.5)	91 (76.5)	119 (18)
25–30	23 (22.3)	80 (77.7)	103 (16)
> 30	34 (11.5)	261 (88.5)	295 (45)
Marital status			
Single	96 (19.6)	394 (80.4)	490 (75)
Married	24 (14.7)	139 (85.3)	163 (25)
Education (years)			
≤ 12	36 (23.5)	117 (76.5)	153 (24)
> 12	84 (16.8)	416 (83.2)	500 (76)
Condom use**			
Always use	49 (13.7)	309 (86.3)	358 (55)
Sometimes/never use	71 (24.1)	224 (75.9)	295 (45)
Clinical symptoms*			
Asymptomatic	47(14.0)	288 (86.0)	335 (51)
Symptomatic	73 (23.0)	245 (77.0)	318 (49)
Burning urination**	*28 (34.6)*	*53 (65.4)*	*81*
*Genital pain/swelling*	*16 (15.8)*	*85 (84.2)*	*101*
Urethral discharge**	*35 (37.2)*	*59 (62.8)*	*94*
*Lower abdominal pain*	*10 (38.5)*	*16 (61.5)*	*26*
*Genital skin rash/ulceration*	*11 (9)*	*96 (18)*	*107*
*Vaginal discharge*^a**^	*12 (35.3)*	*22 (64.7)*	*34*
Sexual orientation			
Heterosexual	107 (18.2)	480 (81.8)	587 (90)
Homosexual/bisexual	13 (20.0)	53 (80.0)	66 (10)
Laboratory diagnoses			
N. gonorrhoeae**	30 (41.0)	43 (58.9)	73 (11)
*T. vaginalis*	4 (66.7)	2 (33.3)	6 (0.9)
*T. pallidum*	5 (13.5)	32 (86.5)	37 (6)
HIV	2 (25.0)	6 (75.0)	8 (1)

Note: Data are the number (%) of patients, except where noted; ^*a *^only the female data were used. ^b ^Percentage in column; *P < 0.05 ; ** P < 0.001 by Chi-square test.

### Risk factors associated with chlamydial infection

Table 1 reveals the results of the univariate analysis and features potential risk factors for chlamydial infection. The overall prevalence of chlamydial infection was 18.4% (95% confidence interval [95% CI] 17.3–19.5%). Prevalence for men and women were 16.7 % (95% CI 15.3–18.0%) and 22.8% (95% CI 17.5–28.1%), respectively. Age group-specific prevalences was 25.7% (95% CI 22.5–28.9%) in < 20 year olds, 23.5% (95% CI 20.3–26.7%) in 20–24 year olds, 22.3% (95% CI 18.9–25.7%) in 25–30 year olds, and 11.5% (95% CI 10.3–12.7%) in > 30 year olds. The prevalence of CT infection among asymptomatic patients was 14.0% (95% CI 12.7–15.3%).

Patients that reported inconsistent condom use had a higher risk for chlamydial infection than patients that reported consistent condom use (P < 0.001). Patients that were symptomatic at the time of testing were more often CT positive than asymptomatic patients (P < 0.05). Patients with *N. gonorrhoeae *infections had higher prevalence of CT infection than those who did not (P < 0.001). There appeared to have been no significant difference with regard to the incidence of infection amongst gender, marital status, education level, and sexual orientation (Table 1).

From the logistic regression model, aged ≤ 30 years (adjusted odds ratio [AOR] = 2.44; 95% confidence interval [CI] = 1.52–3.84; P < 0.001), inconsistent condom use (AOR = 2.01; 95% CI = 1.32–3.06; P < 0.001), being symptomatic at the time of testing (dysuria, urethral discharge) (AOR = 1.84; 95% CI = 1.21–2.80; P < 0.001), and having *N. gonorrhoeae *infection (AOR = 3.82; 95% CI = 2.20–6.58) were significantly, independently and positively associated with the prevalence of chlamydial infection (Table 2).

**Table 2:** Risk factors for chlamydial infection by stepwise logistic regression analysis

Variables	Adjusted Odds Ratio	95% CI
Young age (age ≤ 30 years) (yes/no)	2.44	1.52–3.84*
Inconsistent condom use (yes/no)	2.01	1.32–3.06*
Currently symptomatic (yes/no)	1.84	1.21–2.80*
*N. gonorrhoeae *infection (yes/no)	3.82	2.20–6.58*

CI, confidence interval; dependent variable was *C. trachomatis *(+)/*C. trachomatis *(-); Independent variables available in the model were young age (age ≤ 30 years) (yes/no), inconsistent condom use (yes/no), currently symptomatic (dysuria, urethral discharge) (yes/no), *N. gonorrhoeae *infection (yes/no). Only results of significant variables are presented. *P < 0.001.

## Discussion

Our study identified a relatively high prevalence of chlamydial infection (18.4% overall; range 17.3–19.5%) among patients attending an STD or genitourinary clinic in Taiwan. Risk factors found to be independently associated with chlamydial infection were young age (< 30 years old), inconsistent condom use during sexual intercourse, being symptomatic (dysuria, urethral discharge) at the time of testing, and having gonococcal infection.

Prevalence estimates for Chlamydia range widely between study populations in different settings. Healthcare settings have higher prevalence estimates than population-based studies [14]. In the USA, the prevalence of *C. trachomatis *genital infections amongst sexually active young adults aged 16 to 20 years is 4–17% [15], whereas in the UK [16], the prevalence of *C. trachomatis *infection varies considerably amongst different sub-populations (range 5–13%). Our study' incidence of chlamydia infection in these settings was relatively high.

The findings in this report are subject to at least three limitations. Firstly, the study population was collected from a high risk group. They may not be truly representative of all people aged 17–50 years in Taiwan. Secondly, whilst our STD and urology clinics probably represent the experiences of other STD and urology clinics in the country, the data derived from these clinics may not appropriately reflect the sub-population of CT-infected Taiwanese patients presenting at other community clinics. Third, the methods for identify *T. vaginalis *are less than optimal. However, to our knowledge, it is the first study to be conducted among men and women in this part of the country.

Our study is consistent with those of a previous study [15] which reported a strong association between chlamydia infection and youthful age. Chlamydia was most common among men aged 18–24 years, as others have noted [17,18]. Our study found that men aged ≤ 30 were two to three times more likely to be infected with chlamydia than those aged > 30 years, even after adjusting for urethral symptoms and condom use at last instance of sexual intercourse; age should factor significantly into determining the most likely population of men to be infected with Chlamydia.

Consistent with the previous findings [18], we also found that combining patient self-report symptoms of infection and inconsistent condom use during sex in the past 60 days could identify patients more likely to be infected and could greatly enhance efforts to detect those men regardless of whether they seek care at an STD clinic. In clinical practice, people with symptoms of infection or a history of recent unprotected sex should receive diagnostic testing for Chlamydia. Our data raise the question of whether certain groups of men without symptoms should be routinely tested by virtue of their attendance in the STD clinic. Currently, there are no guidelines on how best to address men who are asymptomatic, much less for men with no symptoms of infection and who have no history of sexual contact with an infected person.

Unsafe sex puts a woman at risk of both sexually transmitted infections and unwanted pregnancy. In this study, patients reporting inconsistent condom use had a higher risk for chlamydial infection than those reporting consistent condom use (AOR = 2.01; 95% CI = 1.32–3.06). These results emphasise that consistent condom use could have a major impact, much as it did in other countries [19,20].

Our study found that concurrent gonococcal infection was significantly associated with chlamydal infection; however, no significant association was found with other STDs (syphilis, trichomoniasis) or HIV infection. One possible explanation is the lower rate (1.2%) of HIV infection in our study population. Another possible explanation for the lack of association between chlamydial infection and other STDs is the high sensitivity of the employed diagnostic *C. trachomatis *PCR assay. This particular assay is capable of detecting infections with very low copy numbers of *C. trachomatis*. These infections would not have been detected by other microbiological assays [21].

## Conclusion

Our study supports the validity of routine testing of all patients with genital symptoms of CT infection and patients who report unprotected sex, regardless of age. While the cost-effectiveness of such an approach requires further study, our study also supports consideration of routine screening among men < 30 years who attend an STD or genitourinary clinic. Our results highlight the notion that the prevention of sexually transmitted disease, including CT, should constitute an important public-health program for Taiwan.

## Competing interests

The author(s) declare that they have no competing interests.

## Authors' contributions

KTC was the lead writer and coordinator of the manuscript, and worked on content development. SCC coordinated data analysis and wrote the background and methods. CCC, LHL and LHT contributed to laboratory work and wrote the results and discussion. All authors read and approved the final manuscript.

## Pre-publication history

The pre-publication history for this paper can be accessed here:


